# The Role of Immune Cells in Moyamoya Disease

**DOI:** 10.3390/brainsci15020137

**Published:** 2025-01-30

**Authors:** Sheng Wang, Qian Jiang, Yuan Liu, Xincheng Zhang, Yimin Huang, Huaqiu Zhang

**Affiliations:** 1 Department of Neurosurgery, Tongji Hospital of Tongji Medical College of Huazhong University of Science and Technology, Jiefang Avenue 1095, Wuhan 430030, China; wangsheng@tjh.tjmu.edu.cn (S.W.); d202382335@hust.edu.cn (Q.J.); liuyuan_upup@163.com (Y.L.); zhangxc3366@163.com (X.Z.); yimin.huang@tjh.tjmu.edu.cn (Y.H.); 2 Hubei Key Laboratory of Neural Injury and Functional Reconstruction, Huazhong University of Science and Technology, Wuhan 430030, China

**Keywords:** moyamoya disease, RNF213, immune cells, macrophage, T cells, dendritic cells, B cells

## Abstract

Moyamoya disease (MMD) is a rare progressive cerebrovascular disorder characterized by the stenosis or occlusion of the terminal segments of the internal carotid arteries, leading to the development of abnormal collateral vascular networks. These networks are a compensatory mechanism for reduced blood flow to the brain. Despite extensive research, the exact etiology of MMD remains unknown, although recent studies suggest that immune system dysfunction plays a critical role in its pathogenesis. In particular, the involvement of immune cells such as T cells, macrophages, and dendritic cells has been increasingly recognized. These immune cells contribute to the inflammatory process and vascular remodeling observed in MMD patients, further complicating the disease’s progression. Inflammation and immune-mediated damage to the vessel walls may accelerate the narrowing and occlusion of arteries, exacerbating ischemic events in the brain. Additionally, studies have revealed that certain genetic and environmental factors can influence immune system activation in MMD, linking these pathways to disease development. This review aims to provide a comprehensive overview of the immune mechanisms at play in MMD, focusing on how immune cells participate in vascular injury and remodeling. Understanding these immunological processes may offer new therapeutic targets to halt or reverse disease progression, potentially leading to more effective treatment strategies for MMD.

## 1. Introduction

MMD is a rare but serious condition predominantly affecting children and young adults, leading to strokes, transient ischemic attacks, and hemorrhagic events [1,2]. The term “moyamoya” originates from a Japanese word that translates to “puff of smoke”, which aptly describes the visual characteristics of abnormal vascular networks observed in angiographic imaging [3]. While genetic factors, particularly mutations in the RNF213 gene, have been implicated, the exact mechanisms underlying the vascular alterations observed in MMD remain incompletely understood [4,5,6]. It has been reported that patients with MMD exhibit not only cerebrovascular lesions but also extracranial manifestations, including coronary artery disease and pulmonary artery stenosis. Notably, these lesions share common pathological features [7]. In line with this observation, the p.R4810K mutation in RNF213 has also been demonstrated to be associated with coronary artery disease [8,9]. However, the low penetrance observed in genetically predisposed individuals implies that an additional ’second hit’ may be required to initiate disease onset. Recent molecular studies have identified RNF213 as a crucial antimicrobial protein with significant roles within the immune system [6,10,11,12,13,14]. Together, this expanding body of molecular and clinical evidence suggests that immune-related responses may serve as secondary triggers for the onset of MMD. This indicates that immune cells could play a crucial role in both the initiation and progression of the disease [15,16,17,18].

This study searched multiple authoritative databases, including PubMed and Web of Science. In PubMed, a search strategy combining both subject headings and free terms was employed to ensure comprehensive coverage of literature relevant to the research topic. For example, search terms such as “immune cells” AND “moyamoya disease” were used. The included literature had to meet the following criteria: first, the research content must be directly related to the function of RNF213, the role of immune cells, and the pathogenesis of MMD; second, the literature must be published within the past 5 years to ensure the timeliness of the information; third, the literature type should primarily consist of original research articles, reviews, and meta-analyses to ensure the reliability and comprehensiveness of the research. The following types of literature were excluded: non-English language papers (unless there was a reliable English translation available); literature based solely on preliminary hypotheses without experimental data or clinical evidence; conference abstracts; and papers for which the full text was inaccessible.

## 2. Overview of Immune Cells Involved in MMD

The current research indicates that various immune cells, including T cells, B cells, macrophage, dendritic cells (DCs), and a range of cytokines, play significant roles in the initiation and progression of MMD [19,20]. These cells contribute to chronic inflammation, vascular remodeling, and endothelial dysfunction, which are all fundamental characteristics of the disease.

### 2.1. T Cells

#### 2.1.1. T Cells and Vascular Aging

Vascular aging refers to the gradual deterioration of the blood vessels over time, a process characterized by endothelial dysfunction, increased arterial stiffness, and a predisposition to cardiovascular diseases such as hypertension and atherosclerosis [21,22]. Aging is associated with changes in the vasculature, including the thickening of the arterial wall, the loss of endothelial function, and the accumulation of extracellular matrix components. Early studies have also found that the smooth muscle cells in moyamoya disease exhibit a senescent state and are impaired in their response to PDGF. This seemingly suggests that vascular aging may be one of the important pathogenic mechanisms of moyamoya disease [23]. The mechanisms underlying the relationship between moyamoya disease and cellular senescence remain unclear, and while research on other diseases has shown that many factors contribute to vascular aging, recent research has identified the immune system, particularly T cells, as key players in this process [24].

T cells are a type of white blood cell that plays a central role in the adaptive immune response. These cells are involved in recognizing and responding to foreign antigens, but they also play a role in tissue remodeling, inflammation, and the regulation of other immune cell types [25]. As the body ages, the immune system undergoes significant changes that impact the function of T cells. This process, known as immunosenescence [26], has profound implications for vascular health. Immunosenescence refers to the gradual decline in immune function that occurs with aging. This process leads to a shift in the balance of immune cell populations, with a reduction in the number and function of regulatory immune cells and an increase in pro-inflammatory cells. The immune system becomes less efficient at responding to infections, and chronic low-grade inflammation—often referred to as “inflammaging”—becomes more prominent. These immune system changes contribute to the pathogenesis of vascular aging [27].

One of the most significant effects of immunosenescence is the altered composition of T cell populations. As individuals age, there is a noticeable increase in the number of effector T cells, particularly Th1 and Th17 cells [28], which are associated with pro-inflammatory responses. In contrast, the number and function of regulatory T cells (Tregs), which help to maintain immune homeostasis and control inflammation, decrease with age [29]. This imbalance between pro-inflammatory effector T cells and anti-inflammatory Tregs is a hallmark of vascular aging and plays a pivotal role in the development of endothelial dysfunction and arterial stiffness [30,31].

Th1 cells are a subset of helper T cells that primarily produce interferon-gamma (IFN-γ), a cytokine that promotes the activation of macrophages and other immune cells. IFN-γ is a potent pro-inflammatory cytokine that has been implicated in various aspects of vascular aging. In particular, IFN-γ promotes endothelial dysfunction by inducing the expression of adhesion molecules on endothelial cells, which facilitates the recruitment of immune cells to the site of injury. Additionally, IFN-γ increases the production of reactive oxygen species (ROS), which further damages the endothelial cells and accelerates the process of vascular aging. Th1 cells also contribute to the thickening of the arterial wall and the development of arterial stiffness. This occurs through the activation of smooth muscle cells, which are responsible for maintaining the structural integrity of the blood vessels. Th1 cells can induce smooth muscle cell proliferation and migration, leading to increased extracellular matrix deposition and the stiffening of the arteries. These processes are hallmark features of vascular aging and contribute to an increased risk of hypertension and atherosclerosis [32]. Th17 cells are another subset of helper T cells that produce interleukin-17 (IL-17), a pro-inflammatory cytokine that plays a central role in the recruitment of neutrophils and macrophages to sites of injury [33]. IL-17 has been shown to contribute to endothelial dysfunction by promoting the release of inflammatory mediators, including cytokines and chemokines. This leads to increased vascular permeability and the accumulation of immune cells in the vascular wall, further exacerbating inflammation and tissue damage [34]. Th17 cells also promote the development of arterial stiffness by inducing the production of matrix metalloproteinases (MMPs), enzymes that degrade the extracellular matrix [35]. The degradation of the extracellular matrix weakens the structural integrity of the blood vessels, making them more prone to damage and contributing to the progression of vascular aging.

Regulatory T cells (Tregs) are a subset of T cells that play a crucial role in maintaining immune homeostasis and preventing excessive inflammation. Tregs are essential for controlling the immune response and preventing autoimmune diseases [36]. They achieve this by secreting anti-inflammatory cytokines, such as interleukin-10 (IL-10) and transforming growth factor-beta (TGF-β) [37], which inhibit the activation of effector T cells and other immune cells. In the context of vascular aging, Tregs play a protective role by suppressing the pro-inflammatory responses that contribute to endothelial dysfunction and arterial stiffness. However, as individuals age, the number and function of Tregs decline, leading to a loss of immune regulation and an increase in chronic low-grade inflammation. This loss of Treg function has been linked to several aspects of vascular aging, including the development of hypertension, atherosclerosis, and increased vascular permeability. One of the key mechanisms by which Tregs protect the vasculature is through the regulation of the immune response to vascular injury. Tregs can inhibit the activation of Th1 and Th17 cells, thereby reducing the production of pro-inflammatory cytokines and preventing excessive tissue damage. Additionally, Tregs promote the resolution of inflammation by inducing the apoptosis of activated immune cells and facilitating tissue repair. In the absence of Tregs, the inflammatory response becomes dysregulated, leading to persistent inflammation and the acceleration of vascular aging.

The presence of senescent cells in the vasculature contributes to the activation of Th1 and Th17 cells, which further exacerbate the inflammatory response. Additionally, senescent cells impair the function of dendritic cells, which are responsible for presenting antigens to T cells. This dysregulation of antigen presentation leads to an exaggerated immune response, further promoting the activation of pro-inflammatory T cells and the progression of vascular aging [38,39].

T cells contribute to endothelial dysfunction by releasing pro-inflammatory cytokines that activate endothelial cells and promote the expression of adhesion molecules. This leads to the recruitment of other immune cells to the site of injury, further exacerbating the inflammatory response. In addition, the production of ROS by activated T cells damages the endothelial cells and impairs their function, accelerating the process of vascular aging [40].

Given the roles played by T cell populations such as Th1, Th17, and Tregs in vascular aging, it can be speculated that these cell populations are likely to have played a role in the occurrence and development of moyamoya disease.

#### 2.1.2. T Cells and Vascular Hypertension

Hypertension, commonly known as high blood pressure, is a significant risk factor for a variety of cardiovascular diseases. It is a multifactorial condition influenced by genetic, environmental, and lifestyle factors. Recent research has highlighted the crucial role of immune cells, particularly T cells, in the development and progression of hypertension [41,42]. T cells are activated in response to various stimuli, including infections, tissue injury, and chronic inflammation. In the context of hypertension, the activation of T cells leads to the release of pro-inflammatory cytokines and the recruitment of other immune cells to the vasculature, contributing to endothelial dysfunction and increased vascular resistance.

The two main subsets of T cells involved in hypertension are effector T cells, such as Th1 and Th17 cells, and regulatory T cells (Tregs). The balance between these subsets plays a crucial role in the development of hypertension, with an increase in pro-inflammatory effector T cells and a decrease in Tregs, contributing to the onset and progression of high blood pressure [42,43].

Th1 cells are a subset of helper T cells that primarily produce interferon-gamma (IFN-γ), a potent pro-inflammatory cytokine [44]. IFN-γ has been implicated in the pathogenesis of hypertension through several mechanisms. First, IFN-γ promotes the activation of endothelial cells, which increases the expression of adhesion molecules and facilitates the recruitment of immune cells to the site of vascular injury. This leads to the accumulation of immune cells in the vascular wall, exacerbating the inflammatory response and contributing to endothelial dysfunction. Additionally, IFN-γ stimulates the production of reactive oxygen species (ROS), which damage the endothelium and impair its ability to regulate vascular tone, leading to increased vascular resistance and elevated blood pressure. Another important effect of IFN-γ is its ability to promote the proliferation and migration of smooth muscle cells in the arterial wall. This process, known as vascular remodeling, contributes to the thickening of the arterial walls and the development of arterial stiffness, both of which are hallmark features of hypertension. Furthermore, IFN-γ can activate the renin–angiotensin–aldosterone system (RAAS), a key regulator of blood pressure. The activation of RAAS leads to increased levels of angiotensin II, a potent vasoconstrictor, which further raises blood pressure and exacerbates hypertension [45,46].

Th17 cells are another subset of T cells that play a significant role in the pathogenesis of hypertension. Th17 cells are characterized by their production of IL-17. IL-17 has been shown to contribute to hypertension by promoting endothelial dysfunction and increasing vascular permeability. By recruiting immune cells to the vasculature, IL-17 amplifies the inflammatory response, leading to further damage of the endothelial cells and an increase in vascular resistance. Th17 cells also contribute to hypertension through their ability to produce MMPs, enzymes that degrade the extracellular matrix. The degradation of the extracellular matrix weakens the structural integrity of the blood vessels and promotes arterial remodeling, leading to increased stiffness and further elevation of blood pressure. In addition, IL-17 has been shown to induce the expression of adhesion molecules on endothelial cells, facilitating the recruitment of leukocytes and exacerbating the inflammatory response in the vasculature [47,48].

Tregs are essential for controlling the activation of effector T cells and preventing the development of autoimmune diseases. In the context of hypertension, Tregs play a protective role by suppressing the activation of pro-inflammatory effector T cells and promoting the resolution of inflammation. However, as individuals age or in the presence of chronic disease, the number and function of Tregs decline, leading to an imbalance between pro-inflammatory effector T cells and anti-inflammatory Tregs. The reduction in Treg function is a key contributor to the development and progression of hypertension. In animal models of hypertension, depletion of Tregs has been shown to exacerbate high blood pressure and increase vascular inflammation. Conversely, the expansion of Tregs can reduce blood pressure and improve vascular health by suppressing the activation of Th1 and Th17 cells and promoting the resolution of inflammation [49,50,51,52].

Vascular resistance is a key determinant of blood pressure, and its regulation is tightly controlled by the balance between vasodilatory and vasoconstrictive factors. In hypertensive individuals, vascular resistance is often elevated due to the thickening of the arterial walls and the loss of endothelial function. T cells contribute to increased vascular resistance through their ability to promote vascular remodeling, smooth muscle cell proliferation, and extracellular matrix deposition. The inflammatory cytokines produced by activated T cells, including IFN-γ and IL-17, play a critical role in promoting vascular remodeling. These cytokines stimulate the proliferation and migration of smooth muscle cells, leading to the thickening of the arterial wall and the development of arterial stiffness. Additionally, T cells contribute to the deposition of extracellular matrix components such as collagen and elastin, which further stiffen the arteries and increase vascular resistance. The increased vascular resistance leads to elevated blood pressure, creating a vicious cycle that exacerbates hypertension and contributes to the progression of cardiovascular disease [53,54].

In summary, in hypertension, vascular wall damage caused by long-term hypertension activates the immune response. The infiltration of T cells and the release of cytokines promote vascular inflammatory responses and remodeling, further exacerbating vascular sclerosis and stenosis. In contrast, the pathogenesis of MMD primarily involves the gradual narrowing or occlusion of the arteries at the base of the brain, leading to reduced blood flow. In this process, T cells may participate in local inflammatory responses through immune reactions, causing endothelial damage and vascular wall remodeling, thereby promoting the formation of collateral vessels. The activation of T cells and the secretion of cytokines may exacerbate vascular wall damage and pathology. Although the role of T cells in moyamoya disease is not yet fully understood, it is clear that immune responses play a potential role in the pathogenesis of this disease.

#### 2.1.3. T Cells and Vascular Inflammation

In addition to investigating various forms of cancer, research on T cells and vascular inflammation primarily addresses the issues of hypertension and atherosclerosis. T. J. Guzik demonstrated that mice deficient in lymphocytes (Rag^−/−^ mice) do not exhibit hypertension or adverse vascular remodeling in response to angiotensin II (Ang II). The reconstitution of T cells in Rag^−/−^ mice at the onset of Ang II infusion effectively reverses this phenotype, leading to the development of hypertension and vascular inflammation. This finding underscores the pivotal role of T cells in regulating both hypertension and vascular inflammation [55]. Mechanistically, CXCR3 facilitates the migration of CD4+ T cells to the heart under conditions of pressure overload, thereby exacerbating cardiac inflammation, fibrosis, and dysfunction [56].

T cells are activated in response to antigens presented by antigen-presenting cells (APCs), such as dendritic cells, within hypertensive environments. This activation results in the generation of effector T cell populations, including Th1 and Th17 subsets, which secrete pro-inflammatory cytokines such as IFN-γ and IL-17 [57,58]. Activated T cells secrete cytokines that potentiate the inflammatory response. For example, IFN-γ plays a crucial role in activating macrophage, whereas IL-17 is involved in the recruitment of neutrophils and enhances the production of reactive oxygen species (ROS) by vascular cells [57]. These cytokines disrupt endothelial function, thereby promoting vascular stiffness and remodeling. T cells play a significant role in endothelial dysfunction by inducing the production of reactive oxygen species (ROS) and reducing the availability of nitric oxide (NO), which impairs vasodilation. Consequently, this leads to an increase in vascular resistance [42].

#### 2.1.4. T Cell and MMD: Histopathological Evidence

Histological analyses of cerebral arteries from patients with MMD have consistently demonstrated significant infiltration of T cells within the vascular lesions [59]. Studies employing machine learning methodologies have demonstrated that, in comparison to healthy control subjects, MMD patients exhibit a significantly higher abundance of NKT cells alongside a markedly lower abundance of Th2 cells within their vascular system. This phenomenon suggests the presence of a pro-inflammatory environment in individuals afflicted with MMD [60].

It has indicated that patients with MMD exhibit lower levels of CD8+ T cells and resting memory CD4+ T cells. Furthermore, there was no significant difference observed in the numbers of immune infiltrating cells that are increased versus those that are decreased [61]. Given the contradictory findings in both studies, additional evidence is essential to elucidate the role of T cells in the initiation and progression of MMD.

#### 2.1.5. T Cell and MMD: Peripheral Immune Profiles

**Figure 1:**
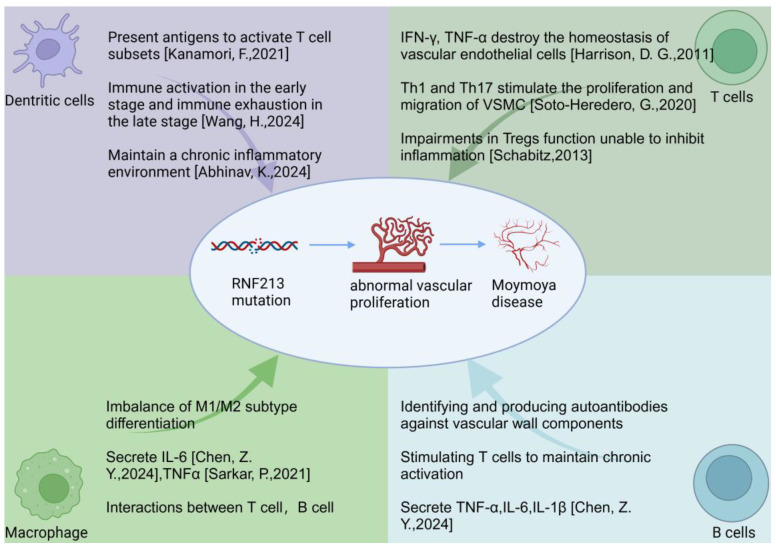
The potential role of immune cells in the progression of MMD. References: Kanamori, F., 2021 [65], Wang, H., 2024 [66], Abhinav, K., 2024 [67], Harrison, D. G., 2011 [57], Soto-Heredero, G., 2020 [28], Schabitz, 2013 [62], Chen, Z. Y., 2024 [68], Sarkar, P., 2021 [69]. Created in BioRender (https://BioRender.com).

Peripheral blood analyses of patients with MMD have demonstrated significant alterations in T cell subsets when compared to healthy controls. Immune dysregulation observed in MMD patients is characterized by notable changes in T cell populations, including a decrease in effector T cells and an increase in Tregs [16]. The research findings strongly indicate that the peripheral immune system is suppressed. Subsequent results demonstrate a significant increase in the proportion of vulnerable regulatory Tregs, defined as those expressing Foxp3 but exhibiting lower levels of inhibitory factors such as IL2RA. In contrast, there is a decrease in the proportion of stable Tregs, which are characterized by their expression of Foxp3 and their inhibitory functions. This suggests that the increase in Tregs does not effectively suppress inflammation, ultimately contributing to chronic inflammatory states in patients with coronary artery vasculitis. Furthermore, impairments in Treg function have been linked to the onset and progression of cerebrovascular diseases [62]. This observation aligns with the findings of Guo et al., who reported a significant increase in pro-inflammatory and immunosuppressive capacities among patients with MMD. This increase was accompanied by a notable decrease in anti-inflammatory and immunomodulatory capacities [63]. Moreover, Tregs secrete angiogenic factors that facilitate uncontrolled angiogenesis and contribute to the incomplete vascular development observed in tumors [64]. This may suggest that Tregs may be involved in angiogenesis in MMD. Overall, in addition to Treg cells, activated T cells contribute to the progression of MMD by secreting various pro-inflammatory cytokines, such as IFN-γ and TNF-α (Figure 1). These cytokines disrupt the stability of vascular endothelial cells, induce endothelial dysfunction, and consequently promote the formation and progression of arterial stenosis. Cytokines secreted by Th1 and Th17 cells can stimulate both the proliferation and migration of vascular smooth muscle cells. Although the specific mechanisms underlying these processes require further investigation and confirmation, targeting T cells and related immune pathways may offer new strategies for treating moyamoya disease.

### 2.2. B Cells

#### 2.2.1. Autoantibodies and Vascular Diseases

Vasculitis refers to the inflammation of blood vessels, which can affect vessels of any size and lead to a wide array of symptoms, depending on the specific vessels involved. A prominent feature of many forms of vasculitis is the presence of autoantibodies that contribute to the inflammatory process. These autoantibodies often target components of the vessel wall, such as endothelial cells, smooth muscle cells, and the extracellular matrix, causing damage and initiating inflammatory cascades. Anti-endothelial cell antibodies are one class of autoantibodies that are frequently found in vasculitis. These antibodies can bind directly to endothelial cells, leading to endothelial cell activation and dysfunction, which is a hallmark of vasculitis. The resulting endothelial injury promotes the infiltration of inflammatory cells, which further aggravates the vascular damage. Anti-neutrophil cytoplasmic antibodies (ANCAs) are another important group of autoantibodies, particularly in diseases like granulomatosis with polyangiitis and microscopic polyangiitis. ANCAs specifically target neutrophils, causing them to release pro-inflammatory cytokines and enzymes that damage the vessel walls. This results in vasculitis-associated tissue destruction and the characteristic necrotizing lesions seen in affected organs [70,71]. The hypothesis postulating that chronic vessel inflammation is the cause of MMD conflicts with the acute deterioration phases of MMD. This conflict is vividly illustrated by MRI findings. Notably, concentric vessel wall enhancement can be detected prior to the appearance of both clinical symptoms and imaging-based evidence of deterioration [23,72,73].

Atherosclerosis is characterized by the accumulation of fatty plaques in the arterial wall. It is the main cause of cardiovascular diseases and can lead to conditions like coronary artery disease and stroke. Traditionally, it was thought to be caused by lipid accumulation and endothelial injury. However, increasing evidence now shows that autoimmunity is essential in its pathogenesis [74]. Take anti-phospholipid antibodies as an example. They target phospholipids in the endothelial cell membrane, causing endothelial dysfunction, increased platelet aggregation, and enhanced thrombus formation, thus promoting the formation of atherosclerotic plaques. In addition, specific autoantibodies against oxidized low-density lipoprotein (ox-LDL) found in patients can enhance the uptake of ox-LDL by macrophages, leading to the formation of foam cells, which promote inflammation and the progression of atherosclerosis. Anti-cardiolipin antibodies are associated with cardiovascular events in patients with antiphospholipid syndrome, increasing the risk of thrombosis, causing vascular occlusion, and leading to related complications [75].

Recent studies have provided evidence that autoantibodies can contribute to the development and maintenance of hypertension [76]. While the precise mechanisms are not yet fully understood, several key pathways through which autoantibodies can influence blood pressure regulation and vascular function have been identified. Anti-angiotensin II type 1 receptor (AT1R) antibodies are one of the most studied autoantibodies in the context of hypertension. These antibodies bind to the AT1 receptor, which is part of the renin–angiotensin system (RAS). The binding of AT1R antibodies can mimic the effects of angiotensin II, leading to vasoconstriction, increased aldosterone secretion, and sodium retention, all of which contribute to elevated blood pressure. In animal models, the administration of these antibodies has been shown to induce hypertension [77], and the presence of these antibodies in human hypertensive patients has been associated with more severe disease. Anti-endothelin-1 antibodies are another group of autoantibodies linked to hypertension. Endothelin-1 is a potent vasoconstrictor that plays a significant role in regulating vascular tone. Autoantibodies targeting endothelin-1 can disrupt its normal function, leading to sustained vasoconstriction and an increase in blood pressure [78]. These antibodies are thought to contribute to the vascular dysfunction observed in some forms of secondary hypertension, particularly in patients with kidney disease or autoimmune conditions.

The mechanisms by which autoantibodies contribute to vascular damage are diverse, but they generally involve immune complex formation, endothelial dysfunction, and the promotion of inflammation. The binding of autoantibodies to self-antigens on endothelial cells or the extracellular matrix can result in the formation of immune complexes, which activate complement and recruit immune cells such as neutrophils and macrophages. These immune cells release cytokines, proteases, and ROS that damage the vascular wall, leading to inflammation, tissue injury, and fibrosis. Autoantibodies also promote endothelial dysfunction by interfering with the normal function of endothelial cells [79,80,81,82].

There are significant differences in immunocyte-related pathogenesis between MMD and vasculitis. The immune response in MMD is mainly local. T cells may respond to cerebral hypoperfusion by promoting the remodeling of blood vessel walls and the formation of collateral vessels. However, this immune response is not caused by systemic immune abnormalities and usually does not trigger widespread vascular inflammation. In contrast, vasculitis is a systemic disease caused by immune system abnormalities. Immune cells such as T cells and B cells directly attack the vascular endothelium, leading to widespread vascular inflammation and damage, accompanied by multi-organ damage, and presenting as a systemic immune response. The differences between the two lie in the local or systemic nature of the immune response, as well as the mechanisms of vascular injury and clinical manifestations. Not only that, steroids can be used to treat vasculitis, but there are currently no guidelines for the treatment of MMD with steroids [83].

#### 2.2.2. B Cells and Vascular Inflammation

B cells play a multifaceted role in vascular inflammation, functioning both to promote and regulate immune responses within arterial plaques. Pro-inflammatory B cell subsets, particularly B2 cells, contribute to the progression of atherosclerosis by producing antibodies against oxidized low-density lipoprotein and secreting pro-inflammatory cytokines such as IL-6 and TNFα. These mediators exacerbate endothelial dysfunction and attract additional immune cells to the vessel wall [84,85,86]. Conversely, regulatory B cells (Bregs) produce anti-inflammatory cytokines such as IL-10, which help reduce inflammation and stabilize plaques [55,87,88]. The equilibrium among the various subsets of B cells is pivotal in determining the degree of vascular inflammation and the progression of atherosclerotic plaques. Furthermore, B cells engage with other immune cell types, such as T cells and DCs (Figure 1), to either amplify or inhibit inflammatory pathways. This interaction further modulates the chronic inflammatory environment.

#### 2.2.3. B Cells and Immune Dysregulation in MMD

In MMD, inflammation and immune system activation are pivotal characteristics. Although the primary etiology of the disease remains unclear, immune dysregulation may play a significant role in its pathogenesis. B cells, as crucial components of immune responses, may contribute to this process by producing pro-inflammatory cytokines and antibodies that exacerbate inflammation. Inflammatory cytokines such as TNF-α, IL-6, and IL-1β [68] are known to be elevated in patients with MMD, which can influence B cell function [89], leading to the activation of autoreactive B cells and the production of antibodies targeting vascular tissues. This contributes to endothelial damage and vascular remodeling. By presenting autoantigens to autoreactive T cells and releasing pro-inflammatory molecules, B cells play a crucial role in the development and progression of chronic inflammatory diseases. Research has shown that patients with MMD exhibit an increased percentage of B cells, along with enhanced migratory and adhesive capabilities of these cells. Additionally, there is a notable elevation in the expression levels of CXCR3, CX3CR1, CD11b, and CD11c. These findings indicate a strong association between B cells and the pathogenesis of MMD [16].

#### 2.2.4. Autoimmune Responses and B Cell Involvement

There is an increasing interest in the role of autoimmune mechanisms in the pathogenesis of MMD. Research has indicated that B cells may contribute to the autoimmune-like responses observed in MMD [15,90]. B cells have the capacity to produce autoantibodies that may target endothelial cells or other vascular structures, thereby contributing to the damage and remodeling observed in MMD. Thyroid autoantibodies can function as immunoinducers in MMD patients who do not possess the RNF213 p.R4810K variant of the susceptibility gene. Furthermore, the coexistence of both thyroid autoantibodies and genetic variants appears to exacerbate the disease, indicating a potential synergistic effect between these factors. This suggests that thyroid autoantibodies are secreted by thyroid B cells, highlighting a significant correlation between autoimmune responses mediated by B cells and MMD [91]. Thyroid autoantibodies may also contribute to vascular damage, which seems to be an immune-mediated factor that exacerbates symptoms such as bleeding in patients with MMD [92]. These autoantibodies may arise from a disrupted immune tolerance mechanism, wherein B cells are unable to effectively differentiate between self- and non-self-antigens. The presence of autoreactive B cells has the potential to exacerbate vascular inflammation and contribute to the pathogenesis of MMD.

We have previously highlighted the pivotal role of T cells in the immune response; however, B cells also serve as APCs. B cells are capable of capturing and processing antigens from the vascular environment, subsequently presenting them to T cells to facilitate their activation. This mechanism may enhance the immune response, potentially resulting in increased inflammation and damage to endothelial cells. The antigen presentation mediated by B cells could elucidate the chronic immune activation observed in MMD, particularly within the context of ongoing blood–brain barrier disruption and persistent vascular inflammation.

In MMD, B cells may contribute to the progression of the condition through various immune mechanisms. They are capable of recognizing and producing autoantibodies against components of the vascular wall, leading to the formation of immune complexes that trigger complement activation and inflammatory responses. As highly efficient APCs, B cells can assist T cells in sustaining chronic activation and broadening the scope of inflammation. Concurrently, pro-inflammatory subpopulations of B cells secrete cytokines that exacerbate local inflammation, while regulatory B cells with compromised function further destabilize the immune microenvironment. Through these synergistic effects, B cells have the potential to worsen vascular damage, promote smooth muscle cell proliferation, and facilitate vascular remodeling in moyamoya disease. Consequently, they play a significant role in advancing both the onset and progression of this disorder.

### 2.3. Macrophage

#### 2.3.1. Macrophage and Vascular Inflammation

Macrophage play a central and dynamic role in vascular inflammation, serving as key immune effector cells and regulators of the vascular wall environment. They originate from circulating monocytes and infiltrate the endothelial layer in response to endothelial dysfunction and inflammatory signaling. In inflamed vessels, macrophage differentiate into various phenotypes—from pro-inflammatory cells driving inflammation to more favorable repair-promoting and inflammation-resolving cell types. Their interactions with modified lipoproteins, cytokines, chemokines, and the production of reactive oxygen species, as well as their influence on other vascular cells, collectively shape the inflammatory environment of the vessel. Therefore, targeting the activity and polarization of macrophage is a promising therapeutic strategy for alleviating vascular inflammation [93]. While tightly regulated inflammation is essential for normal angiogenesis, dysregulated inflammation can have significant clinical consequences as it leads to aberrant angiogenesis and impairs vascular function. For instance, inflammatory bowel disease (IBD) is primarily driven by chronic inflammation involving macrophage in the intestinal mucosa. This condition results in the continuous formation of new blood vessels, which increases local vessel density and supports ongoing tissue regeneration in damaged areas [94]. These vessels exhibit a deficiency in pericyte coverage, resulting in inadequate perfusion and leakage, which significantly contributes to the progression of IBD [95]. This condition bears resemblance to the pathological features observed in moyamoya disease, thereby underscoring the critical role of macrophage.

#### 2.3.2. Macrophage Polarization in MMD

Macrophage are highly versatile cells capable of undergoing polarization into two primary phenotypes: M1 macrophage, which are pro-inflammatory, and M2 macrophage, characterized by their anti-inflammatory properties and role in tissue repair. In the context of MMD, it is believed that there exists an imbalance in the polarization of these macrophage populations. Generally, M2 macrophage are associated with tissue repair and wound healing; however, within the framework of MMD, they may also contribute to abnormal tissue remodeling and fibrosis, thereby further impacting vascular architecture. Research has demonstrated that CD163 secreted by M2 macrophage plays a significant role in the onset and progression of MMD [96]. These results suggest that M2 macrophage play an important role in the progression of MMD.

#### 2.3.3. Macrophage Infiltration and Vascular Remodeling

Histological studies have demonstrated the presence of macrophage within the vascular lesions of patients with MMD. These cells are capable of infiltrating both the intima and media layers of the affected arteries. The accumulation of macrophage in the vascular wall can promote vascular inflammation and contribute to endothelial dysfunction, which is a hallmark feature of MMD. Macrophage play a significant role in vascular remodeling, a process that leads to the thickening of arterial walls and the formation of abnormal collateral vessels in MMD. By releasing various cytokines, proteases, and growth factors, macrophage influence the extracellular matrix as well as smooth muscle cells and endothelial cells—key components involved in vascular remodeling [59].

#### 2.3.4. Macrophage and Endothelial Dysfunction

Endothelial dysfunction plays a pivotal role in the pathogenesis of MMD as it results in impaired vascular regulation, diminished blood flow, and subsequent ischemia. Macrophage, particularly the M1 subtype, release cytokines and ROS that can directly inflict damage on endothelial cells. Furthermore, macrophage may facilitate the activation of other immune cells such as T cells and neutrophils, which further aggravate endothelial injury.

Macrophage secrete pro-inflammatory cytokines such as IL-6 [68] and TNF-α [69], which play a crucial role in the pathogenesis of MMD. These cytokines can induce the activation of endothelial cells and promote the expression of adhesion molecules, thereby leading to the recruitment of additional immune cells to the site of injury. Additionally, the insufficient secretion of IL-10 by M2 macrophage adversely affects the differentiation of endothelial progenitor cells, impairing angiogenesis and contributing to the progression of MMD [97].

In MMD, macrophages may play a multifaceted role in the progression of the condition through various mechanisms. They accumulate at sites of endothelial damage and differentiate into pro-inflammatory subtypes, secreting an array of cytokines, chemokines, and growth factors that attract additional immune cells to infiltrate the affected areas while simultaneously stimulating vascular smooth muscle cell proliferation and remodeling. Furthermore, macrophages are involved in clearing cellular debris and releasing matrix metalloproteinases that modify the structure and mechanical properties of the vessel wall, thereby promoting the formation of abnormal collateral circulation. These activities, along with macrophages’ involvement in regulating the coordinated actions of T cells, B cells, and other immune components within the immune network, contribute to both maintaining and exacerbating the inflammatory state as well as pathological progression associated with moyamoya disease.

### 2.4. Dendritic Cells

#### 2.4.1. Dendritic Cells and Vascular Inflammation

DCs play a pivotal role in vascular inflammation, acting as key mediators between oxidative stress and immune activation, particularly through their interactions with isoLGs. IsoLGs are highly reactive lipid oxidation products that modify proteins within the vascular environment, generating novel antigens that are efficiently recognized and processed by dendritic cells.

Upon encountering these isoLG-modified antigens, dendritic cells undergo activation and maturation, thereby enhancing their capacity to present these antigens to T cells and secrete pro-inflammatory cytokines. This activation fosters a robust immune response that perpetuates inflammation in vascular tissues, ultimately leading to endothelial dysfunction and facilitating structural and functional alterations associated with hypertension.

Consequently, dendritic cells serve as a critical link between oxidized lipid modifications and ongoing inflammatory processes, highlighting their significance in the pathogenesis of cardiovascular disease [98].

#### 2.4.2. Dendritic Cells in Inflammation and Vascular Remodeling in MMD

DCs play a crucial role in the initiation of the immune response by capturing and processing antigens, which subsequently leads to the activation of naive T cells. In MMD, chronic inflammation is a defining characteristic. By responding to inflammatory signals, DCs may facilitate T cell activation and contribute to the ongoing inflammatory processes. The persistent state of inflammation observed in MMD is believed to be partially responsible for vascular remodeling, encompassing endothelial dysfunction, the proliferation of smooth muscle cells, and the narrowing of blood vessels [65,67].

DCs in MMD may capture autoantigens or microbial components from the vascular environment, thereby triggering an adaptive immune response. These activated T cells subsequently release pro-inflammatory cytokines, which can further exacerbate vascular damage and contribute to the characteristic stenosis and occlusion of arteries associated with MMD [99].

#### 2.4.3. Dendritic Cells and Immune Dysregulation in MMD

Two primary subtypes of DCs are widely acknowledged: conventional dendritic cells (cDCs) and plasmacytoid dendritic cells (pDCs). cDCs play a pivotal role in presenting antigens to T cells, whereas pDCs are primarily involved in the production of type I interferons, particularly during viral responses. Investigating the roles of DC subtypes in MMD may provide valuable insights into how these cells contribute to the chronic inflammatory milieu. We hypothesized that DCs, through their interactions with T cells, could be amplifying immune responses that result in persistent endothelial damage and subsequent vascular remodeling.

**Figure 2:**
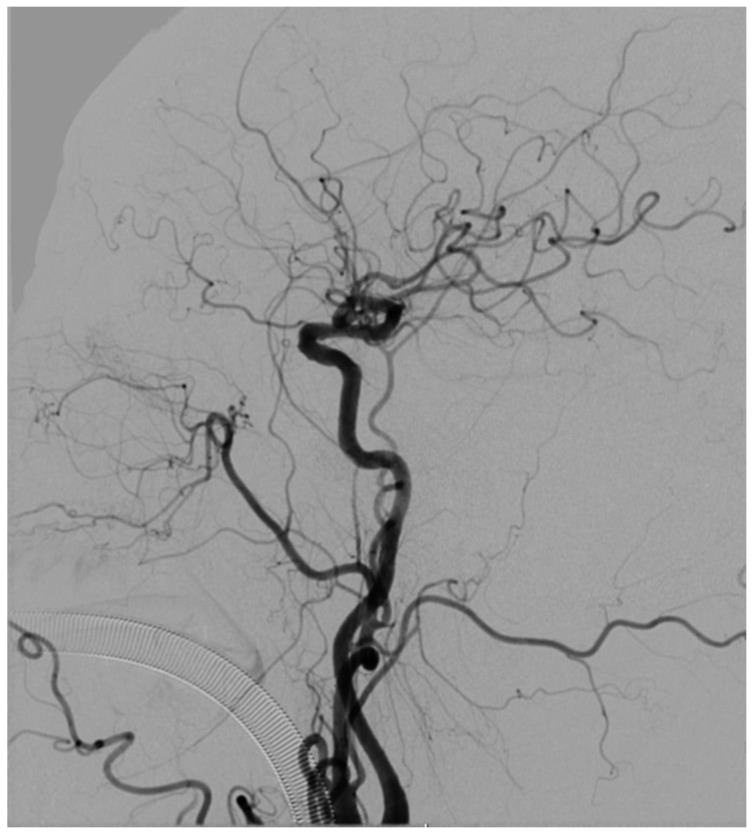
Typical DSA images of MMD.

cyTOF analyses of peripheral blood mononuclear cells (PBMC) from early and late stages of MMD confirmed that the early MMD cohort exhibited an increase in non-canonical monocytes compared to those with advanced MMD. The Suzuki stage of moyamoya disease describes disease progression and cerebral angiography. (Figure 2) In Stage I, there’s stenosis at the internal carotid artery bifurcation with no abnormal vessels. In Stage II, moyamoya vessels emerge. They increase in Stage III, forming a dense network. In Stage IV, they start to decrease, and the decrease is significant in Stage V. By Stage VI, moyamoya vessels vanish, and the anterior and middle cerebral arteries, along with the internal carotid artery’s terminal end, are completely occluded [66]; there is also a noted decrease in the proportion of pDCs and monocyte-derived DCs [100]. This indicates that as the disease progresses, the level of inflammation may diminish, resulting in a relative decrease in these cell types. A reduction in the proportion of DCs could lead to impaired antigen presentation within the body and, during the later stages of MMD, may be linked to immune cell exhaustion. Conversely, in MMD, the potential overactivation of DCs might result in the excessive activation of T cells, thereby promoting chronic inflammation that accelerates vascular changes. In MMD, research has indicated alterations in the immune cell profile, characterized by an increase in pro-inflammatory cytokines and specific subtypes of T cells, potentially driven by DCs. DCs may play a pivotal role in this process by facilitating the differentiation of naive T cells into Th1 and Th17 subsets, which are linked to chronic inflammation and tissue damage.

In MMD, DCs may play a crucial role in the pathogenesis through their efficient antigen-presenting function. This function facilitates the delivery of vascular wall injury or potential self-antigens to T cells, thereby triggering and sustaining chronic inflammation and immune responses. Concurrently, DCs enhance the pro-inflammatory characteristics of the vascular microenvironment by secreting cytokines and chemokines, as well as interacting with other immune cells, thus establishing a connection between innate and adaptive immunity. Moreover, DCs may indirectly influence the phenotypic conversion and proliferation of endothelial and smooth muscle cells, driving vascular remodeling and contributing to the formation of abnormal collateral vessel networks. These synergistic effects position DCs as potentially pivotal players in the pathogenesis of moyamoya disease.

### 2.5. Others

In addition, various immune cells have been demonstrated to play a role in the progression of MMD. For instance, microglia are implicated in the revascularization process associated with MMD. Through the investigation of blood-induced microglia-like (iMG) cells, the authors discovered that M2 microglia may contribute to the angiogenesis involved in MMD [101]. In addition, several studies have indicated that the expression of complement C3 in MMD is diminished and notably lower in patients with advanced Suzuki stages. This observation suggests a potential association between complement C3 and the occurrence as well as progression of MMD [102].

Pro-inflammatory cytokines such as IL-6, TNF-α, IFN-γ, and GM-CSF [103] have been found to be elevated in MMD patients. These cytokines can induce endothelial dysfunction, promote leukocyte recruitment, and enhance the inflammatory milieu within the cerebrovascular system. Additionally, high expression of IL-2, IL-4, IL-5, IL-7, IL-8, IL-9, IL-17, IL-18, IL-22, and IL-23 was found in the cerebrospinal fluid of patients with moyamoya disease [67].

### 2.6. Therapeutic Implications

In terms of treating the common symptoms of MMD, the use of antiplatelet drugs and many other medications focuses on symptom control [104]. The results of a national survey in Japan show that there is a great difference in antiplatelet drug selection among different institutions and no consensus on the treatment method [105]. Some studies suggest that cilostazol may be better than clopidogrel at improving cognitive function in non-surgical adult ischemic moyamoya patients [106], but more research evidence is needed to confirm the effectiveness of antiplatelet drug conservative treatment. Given the vascular cognitive dysfunction caused by MMD [107], acetylcholinesterase inhibitors are usually approved for moderate cognitive benefits [108]. In summary, the effectiveness of MMD drug therapy is still unclear and urgently needs further research [109].

Masaki Ito discovered that mutations in RNF213 are associated with the prognosis of surgery for MMD patients, and patients with mutations have a worse prognosis; these discoveries indicate that modulating the immune response elicited by the RNF213 mutation might become a novel therapeutic approach [110]. Given the close relationship between the RNF213 gene and immune cells, immunotherapy may also be one of the ways to treat MMD in the future. By targeting the key factors or cells in the immune system, especially regulating the functions of immune cells or restoring immune dysregulation, it is possible to effectively alleviate immune-mediated vascular damage and delay the progression of vascular lesions. Therefore, understanding the role of the RNF213 gene in the immune response may provide a new perspective for exploring personalized treatment options.

### 2.7. Challenges and Future Directions

Although the association between immune cells and MMD is compelling, several challenges remain. First, MMD presents with a variety of clinical manifestations and genetic backgrounds, necessitating personalized treatment approaches tailored to individual circumstances. Second, the mechanisms underlying immune cell infiltration in MMD are not yet fully understood; further research is required to elucidate the precise roles that immune cells play in the vascular changes associated with MMD. Moreover, there is currently no stable experimental model for MMD. To advance therapeutic research on this condition, reliable animal models that accurately reflect the immunological and vascular characteristics of human MMD are essential.

## 3. Potential Mechanisms Linking Immune Cells to MMD Pathogenesis

The interaction between immune cells and vascular cells is a critical aspect of MMD pathogenesis. Several mechanisms have been proposed:

### 3.1. Chronic Inflammation

Persistent immune activation results in a chronic inflammatory state within the cerebral vasculature, which promotes endothelial injury and the proliferation of smooth muscle cells. This chronic inflammation is driven by the sustained activation of the immune system, leading to prolonged immune responses that establish a persistent inflammatory condition within the cerebrovascular system. In this context, there is a continuous infiltration of immune cells into the vessel wall, accompanied by the release of substantial amounts of pro-inflammatory cytokines and chemokines. These factors not only exacerbate local immune responses but also directly inflict damage on the endothelial cells lining the blood vessels [5].

### 3.2. Endothelial Damage

Endothelial cells constitute the primary cellular layer of the vascular inner wall and play a crucial role in regulating vessel permeability, blood flow, as well as vascular constriction and dilation. Chronic inflammation leads to endothelial cell damage and functional impairment through the activation of immune cells, particularly macrophage and T cells [111].

Factors secreted by these immune cells, such as TNF-α, IL-1β, and IL-6, can activate endothelial cells, resulting in the apoptosis, increased permeability, and upregulation of adhesion molecules. These alterations facilitate the translocation of immune cells and other inflammatory mediators across the endothelial barrier, thereby exacerbating the local inflammatory response.

### 3.3. Smooth Muscle Cell Proliferation

After endothelial cell damage, the smooth muscle cells within the vessel wall undergo proliferation and migration, contributing to the thickening of the vessel wall. This phenomenon is a consequence of angiogenesis initiated by chronic inflammation. The proliferation of smooth muscle cells constitutes a component of the vascular repair response aimed at restoring damaged vessel walls; however, excessive proliferation and collagen deposition can result in the narrowing of the vessel lumen, ultimately exacerbating vascular stiffness and functional impairment [59]. Studies have shown that the loss of RNF213 function may lead to enhanced proliferation, migration, and lumen-forming capabilities of endothelial cells via the Hippo pathway, further mediating the pathogenesis of MMD [112].

The proliferation of smooth muscle cells and the deposition of extracellular matrix contribute to a phenomenon known as vascular remodeling. This process results in an increase in the thickness of the vessel wall, leading to a narrowing of the vessel lumen and a subsequent reduction in blood flow. Such alterations are particularly critical in cerebrovascular diseases, where the prolonged restriction of blood flow can result in brain ischemia, ultimately precipitating ischemic stroke or other cerebrovascular lesions.

### 3.4. Autoimmunity

Autoantibodies produced by B cells may target endothelial antigens, resulting in immune-mediated vascular damage and subsequent stenosis. Autoimmunity refers to the phenomenon wherein the immune system erroneously attacks normal cells and tissues within the body, typically due to self-antibodies generated by B cells. In autoimmune diseases, B cells secrete self-antibodies that specifically recognize and attack antigens present in self-tissues. The production of these self-antibodies is not only a component of the immune response but can also lead to significant vascular damage, culminating in vascular narrowing [113,114].

Vascular damage caused by autoantibodies is a common feature of many immune-related diseases. For example, in certain types of autoimmune diseases, such as systemic lupus erythematosus [115] and antiphospholipid syndrome [116], autoantibodies attack endothelial cells in the blood vessels, leading to chronic inflammation, vascular damage, and ultimately, vessel narrowing. This immune response is not limited to a local area but may cause damage throughout the body, affecting the function of multiple organs.

### 3.5. Cytokine-Mediated Effects

Elevated levels of cytokines can induce oxidative stress, lead to apoptosis in endothelial cells, and disrupt the regulation of angiogenic factors, thereby contributing to abnormal vessel formation. The impact of cytokine-mediated responses pertains to how an excessive increase in cytokines—particularly pro-inflammatory cytokines—during inflammatory processes can alter the structure and function of blood vessels through various mechanisms. Cytokines are signaling molecules released by the immune system that play a crucial role in regulating immune responses, vascular function, and tissue repair. In the context of vascular diseases, elevated pro-inflammatory cytokines not only activate the immune response but also directly inflict damage on endothelial cells, adversely affecting both the health and functionality of blood vessels.

In MMD, the levels of pro-inflammatory cytokines such as TNF-α, IL-1β, and IL-6 are significantly elevated [117,118]. These cytokines can activate various signaling pathways and affect the function of endothelial cells. Long-term elevation of cytokines promotes the occurrence of oxidative stress, further aggravating endothelial cell damage and inflammatory responses.

Elevated cytokines activate the oxidative stress pathway and increase the production of ROS. ROS accumulate in cells and cause oxidative damage to endothelial cells [119], destroying cell membranes, proteins, and DNA. Oxidative stress not only causes endothelial cell damage but also activates downstream inflammatory responses, further exacerbating damage to the vessel wall [120]. Endothelial cell damage leads to increased permeability of the vessel wall, allowing immune cells and inflammatory mediators to penetrate more easily, resulting in a persistent inflammatory state.

Cytokines can also induce apoptosis in endothelial cells by modulating apoptosis-related signaling pathways. Under the combined influence of oxidative stress and inflammatory factors, endothelial cells progressively lose their functionality, resulting in structural and functional damage to the vessel wall. The apoptosis of endothelial cells compromises the integrity of the vessel wall and initiates vascular remodeling and stenosis.

### 3.6. Dysregulation of Angiogenic Factors

Elevated cytokines can also disrupt the normal regulation of angiogenic factors (such as VEGF and FGF) in MMD [121]. Angiogenic factors play a crucial role in vascular repair and the formation of new blood vessels. However, under conditions of chronic inflammation and oxidative stress, the expression of these angiogenic factors may become dysregulated, resulting in abnormal angiogenesis. This dysregulation can manifest as poorly developed or structurally aberrant blood vessels, which adversely affect both the functionality and blood flow supply of the vascular system.

Due to the dysregulation of angiogenic factor regulation, abnormalities may arise during the process of angiogenesis. In certain diseases, such as atherosclerosis [122], and pulmonary hypertension [123], elevated levels of inflammatory cytokines and increased oxidative stress can lead to an excessive or aberrant formation of new blood vessels. This abnormal angiogenesis not only fails to effectively enhance blood flow but may also result in vessel wall instability, disordered blood flow patterns, and an increased risk of thrombosis.

### 3.7. Function of RNF213 Gene

Genetic factors, such as mutations in the RNF213 gene, may predispose individuals to heightened immune responses or impaired immune regulation, thereby exacerbating vascular pathology. The RNF213 gene is a critical component that is closely associated with moyamoya disease. Mutations or functional abnormalities within this gene have been strongly linked to the occurrence of cerebrovascular diseases in specific populations. This gene encodes a protein that plays a vital role in vascular development and endothelial cell function. Mutations in the RNF213 gene can result in abnormal vascular development, consequently increasing the risk of cerebral vascular stenosis and inadequate blood flow [13,124,125]. Recent research has pointed out that RNF213 plays a crucial role in maintaining the integrity of the blood–brain barrier. The loss of its function can lead to a downregulation of key junction proteins between endothelial cells, such as PECAM-1, and the abnormal localization of these proteins. This may be one of the early pathological mechanisms of moyamoya disease [126].

Research has demonstrated that mutations in the RNF213 gene not only impact the structure and function of blood vessels but may also lead to the abnormal activation of the immune system. Specifically, RNF213 mutations can cause an individual’s immune response to overreact to certain environmental stimuli [14], such as infections, inflammation, or other intrinsic signals. This excessive immune activation can result in the dysregulation of the immune system, leading to the abnormal infiltration of immune cells—including T cells, B cells, and macrophage—into blood vessels. Consequently, this exacerbates vascular inflammatory responses and contributes to tissue damage [127].

In certain instances, mutations in RNF213 may disrupt the balance of immune regulatory mechanisms. Tregs and inhibitory molecules within the immune system may fail to effectively suppress excessive immune responses. This deficiency in effective immune regulation can hinder the immune system’s ability to accurately recognize and respond to normal physiological conditions, potentially leading to autoimmune reactions. Such dysregulation exacerbates the chronic inflammation of blood vessels, facilitates vascular remodeling, and may result in vascular stenosis as well as thickening of the vascular wall, ultimately worsening vascular pathology [6].

Recent studies have shown that RNF213, as an E3 ubiquitin ligase, can regulate proteins related to the inflammatory response through the ubiquitination process. During the inflammatory response, numerous cytokines and signaling pathways are activated. RNF213 may assist endothelial cells in adapting to the inflammatory environment by regulating the degradation or stabilization of these proteins. These inflammatory signals include cytokines such as TNF-α and IL-1, which play crucial roles in vascular inflammation [13].

In individuals with RNF213 mutations, abnormal responses of the immune system will further promote damage to the vascular endothelium, enhance vascular permeability, and increase the accumulation of immune cells in the vascular wall. Long-term infiltration of immune cells and continuous inflammatory responses can lead to structural changes in the vascular wall, thereby forming narrowed blood vessels and restricting normal blood flow. Meanwhile, immune responses may also exacerbate vascular remodeling through pathways such as activating smooth muscle cells and promoting extracellular matrix deposition, preventing blood vessels from effectively maintaining their normal functions.

## 4. Discussion

MMD is a cerebrovascular disorder with an unclear etiology. Due to the lack of effective cells and animal models, research on immune cells in MMD has been limited, and it was once considered a non-inflammatory vascular disease. However, with the rapid advancement of omics technologies, such as transcriptomic sequencing, single-cell sequencing, and mass cytometry, an increasing number of studies have identified the involvement of immune cells—particularly macrophage, T cells, dendritic cells, and B cells—in the pathogenesis and progression of MMD. Despite these advances, conclusions from different studies are not entirely consistent. For instance, a 2023 study by Zhou et al. used bioinformatics to reveal that MMD is characterized by a higher proportion of eosinophils rather than T cells [61]. In contrast, a study conducted by Ge et al. in 2024 employed single-cell sequencing to investigate circulating immune cells. The findings revealed that immune dysregulation in patients with MMD was characterized by alterations in T cell populations, specifically a decrease in effector T cells and an increase in Tregs [16]. These findings indicate that although immune cell infiltration is involved in MMD, the immune response is highly heterogeneous.

The current research suggests that the progression of MMD may be linked to mutations in the RNF213 gene, which make patients more susceptible to immune cell damage. This, in turn, leads to chronic inflammation and promotes endothelial cell proliferation. A study by Ryosuke Tashiro and colleagues further confirmed that RNF213, as a key pathogenic gene for MMD, is highly expressed in immune cells associated with MMD. The knockout or mutation of the RNF213 gene may disrupt the function of antigen-presenting cells, resulting in abnormal T cell responses. The dysregulation of RNF213 function could lead to the abnormal activation of immune responses, further exacerbating vascular inflammation and damage [99].

These findings suggest that combined immunotherapy could offer a promising therapeutic strategy to improve the prognosis of MMD patients. However, due to the limited research on immunotherapy in MMD, further work is needed to build a more comprehensive immune landscape to better identify potential therapeutic targets.

## 5. Conclusions

MMD is a complex cerebrovascular disorder, with increasing evidence suggesting that immune cells play a critical role in its pathogenesis. The interaction between T lymphocytes, B lymphocytes, macrophage, and pro-inflammatory cytokines contributes to the chronic inflammation, endothelial dysfunction, and abnormal vascular remodeling observed in MMD. Targeting immune pathways holds promise as a therapeutic strategy, although further research is needed to fully elucidate the underlying mechanisms and develop effective treatments. Advancements in this field have the potential to improve clinical outcomes and enhance the quality of life for MMD patients.

## Data Availability

Not applicable.
